# Diverging Trends and Expanding Educational Gaps in Smoking in China

**DOI:** 10.3390/ijerph19084917

**Published:** 2022-04-18

**Authors:** Lei Jin, Lin Tao, Xiangqian Lao

**Affiliations:** 1 Department of Sociology, The Chinese University of Hong Kong, RM 431, Sino Building, Shatin, Hong Kong, China; 2 Department of Sociology, Peking University, Beijing 100871, China; taolin@pku.edu.cn; 3 The Jockey Cub School of Public Health and Primary Care, The Chinese University of Hong Kong, Shatin, Hong Kong, China; xqlao@cuhk.edu.hk

**Keywords:** smoking, social disparities in health, socioeconomic status, life course

## Abstract

Introduction: The male smoking rate in China declined moderately through the 1990s and early 2000s, but the decline has since stagnated. It is unclear why the decline stalled and whether it stalled uniformly across all social strata. Theories that view socioeconomic status as a fundamental cause of health predict that socioeconomic gaps in smoking may widen, but theories emphasizing the cultural context of health behavior cast doubt on the prediction. We investigated changes in the socioeconomic gaps in smoking during recent decades in China. Methods: We applied growth-curve models to examine inter- and intra-cohort changes in socioeconomic gaps in male smoking in China using data from a national longitudinal survey spanning 25 years. Results: We found diverging trends in smoking in men with different education levels among the post-1980 cohorts; for high-education men, smoking participation consistently declined, but for low-education men, the decline stopped and possibly reversed. The stagnation in the decline in overall smoking rate since 2010 was mostly due to the stalling of the decline of smoking among low-education men in the most recent cohorts. The diverging trends were a continuation of a general trend in expanding educational gaps in smoking that emerged in the cohorts born after 1960. Our analysis also identified widening educational gaps over age within each cohort. Conclusion: We identified a long-term widening in educational gaps in smoking in China. An effective way to reduce smoking, social inequality in smoking and possibly health disparities in China is to target the smoking behavior of vulnerable groups.

## 1. Introduction

More than 350 million smokers in China, mostly men, consumed one third of the cigarettes produced in the world [1]. Through the 1990s and early 2000s, the rate of cigarette smoking declined consistently albeit moderately in China [2,3,4], but a few recent studies revealed that in the early 2010s, the declining trend stalled and the rate of smoking remained high [5]. For example, the proportion of current smokers among men went from 59.6% in 1993 to 47.0% in 2008, but remained at 47.2% in 2013 [2,5]. It is still unclear why the decline stalled and whether it stalled uniformly across all social strata. In this paper, we investigate the socioeconomic differences in the trend of smoking behavior during this process, which may help ascertain whether the stalling concentrated in certain groups and help to identify these groups. Different theories have conflicting predictions. Theories that view socioeconomic status as a fundamental cause of health predict widening socioeconomic gaps, as the smoking behavior of individuals with low socioeconomic status (SES) may decline more slowly and the decline may stall earlier than that of their higher-SES peers, whereas theories emphasizing the cultural context of health behavior suggest that the widening gaps may not occur. Further empirical research is therefore needed.

In the research on social determinants of health, the theory of fundamental causes of health emphasizes the abilities of members of socially advantaged groups to more readily adopt health-promoting innovations as they became available, such as effective medical treatments or knowledge about healthful lifestyles [6]. As a result, social disparities in health are maintained even as prevalent health problems changed over time [6]. In the case of smoking, as knowledge about its harms is established, compared with low-SES groups, high-SES groups are more likely to obtain this knowledge and, in addition, to have the resources that allow them to adopt the more healthful practices associated with this knowledge. Findings from empirical research conducted in economically developed societies, such as the USA, largely conformed to the prediction [7]. In the 1950s, as the harmful health effects of smoking came to light, the rate of smoking started to decline, and by the 1990s, the smoking rate stabilized at a low level. However, the decline was much slower and stopped earlier and at a higher level among low-SES groups than high-SES groups, which resulted in stark socioeconomic disparities in smoking behavior that persists to this day [7,8,9,10,11]. As China modernized and the public awareness of the health hazard of smoking grew, it is conceivable that more socially advantaged individuals in China were more likely to abstain from smoking than their less advantaged peers and, as a result, social inequality in smoking behavior became exacerbated.

On the other hand, the research on health lifestyle demonstrated that the practice of health behavior is not only influenced by the availability of knowledge and resources, but also conditioned by the social and cultural context [12]. A small but substantial literature has shown that in China, smoking and cigarettes perform important social functions and embody significant cultural meanings [13,14]. In social and work settings, smoking serves to facilitate social interactions, as men routinely take turns distributing and sharing cigarettes. Packets of expensive cigarettes are highly desirable gifts people give and receive during festivals and celebrations to sustain their social network connections. Moreover, these practices have been found to be prevalent among all social strata, even among medical doctors [15,16,17]. Existing research has also shown that moderate consumption of cigarettes increased employment stability among men, adding further evidence to the legitimacy of smoking and pressure to smoke in the Chinese workplace [18]. In addition, a number of studies revealed the widely held beliefs, among both smokers and non-smokers, that smoking symbolized personal freedom, masculinity and male power, and that smoking was important to social interactions [19,20,21,22]. This body of research not only highlights the difficulty and complexity of tobacco control in China [21,22], but also casts doubt on the predictions of the widening socioeconomic gaps in smoking behavior in recent decades in China.

This paper analyzes data from a longitudinal survey spanning 25 years to assess changes in the smoking behavior of different socioeconomic groups in China. To rigorously assess the changes in the socioeconomic gaps in smoking, we adopt the life course perspective and consider the interplay among age, cohort and social context in the analysis [23]. As China has experienced large-scale social and economic changes in recent decades, cohorts that came to age at different time periods may develop distinct patterns of smoking behavior and the influence of socioeconomic factors on smoking may shift across cohorts [8,24,25]. We investigate the changes in socioeconomic differentials in smoking across cohorts, focusing specifically on whether the socioeconomic gaps in smoking widened across cohorts. In the meantime, smoking behavior is known to change with age [26] and socioeconomic disadvantages may accumulate over the life course, leading to expanding socioeconomic gaps in older age [27]. Therefore, we also examine whether the age trajectories of people with high and low SES diverged, resulting in widening socioeconomic gaps. In this paper, we focus on men’s smoking behavior only, because the smoking rate was generally very low among young and middle-aged women in China [28] and the small number of female smokers made it infeasible to assess the interplay of age, cohort and SES among women.

## 2. Methods

### 2.1. Data Collection

#### 2.1.1. Study Sample

The data are from the Chinese Health and Nutrition Survey (CHNS), a national longitudinal study that recorded detailed information on health lifestyle and socioeconomic conditions. Nine waves of the survey, spanning 25 years from 1991 to 2015 (1991, 1993, 1997, 2001, 2004, 2006, 2009, 2011 and 2015), are used. The characteristics of the respondents in the CHNS are comparable to the national average [29]. The analytical sample includes 11,810 men aged between 22 and 75 years during the study period with 38,072 person-year observations.

This study has been approved by the committee on Survey and Behavioral Research Ethics at the Chinese University of Hong Kong.

#### 2.1.2. Variables

Smoking behavior: We use two indicators: the number of cigarettes a person consumed per day at a particular wave (for nonsmokers, daily cigarette consumption is zero) and whether a person was a current smoker at a particular wave (a respondent is defined as a current smoker if he answered yes to the question “Are you currently smoking” and reported smoking one or more cigarette per day).

Cohort: Respondents are assigned to one of 32 birth cohorts; each cohort spans two birth years (e.g., 1981–82), except for those born before 1925 and those born on or after 1985.

Age: Following the methodological literature on growth-curve models, individuals’ time-varying age is subtracted by the baseline age, which is defined as the youngest age in the cohort to which the individuals belong at the time when the cohort entered the study, so as to minimize the collinearity between age and cohort [25].

SES: We assess two indicators of SES: levels of education and household income. A cohort-specific measure of education is used to define high, medium, and low levels of education. In China, educational opportunities greatly expanded from the older to younger cohorts covered in the survey. Given the extensive imbalance in educational levels among the cohorts, using criteria that are constant across cohorts (e.g., primary school, high school and college) is undesirable because the composition of the groups with the same levels of education may be drastically different across cohorts. For example, in recent cohorts, as the proportion of low-education individuals (e.g., ≤primary-school education) shrank drastically, low-education individuals may become increasingly selected for disadvantaged familial or individual characteristics, which may also predispose them for risk behaviors such as smoking. We may observe a spurious widening of the educational gap in smoking across these cohorts simply because of the selection. We therefore used a cohort-specific measure such that the proportions of respondents categorized as having high, medium and low levels of education are roughly similar across cohorts. The specific definition and distribution of levels of education in each cohort are displayed in Supplementary Text S1. To confirm the robustness of our findings, we used the alternative cohort-constant measure of education in the sensitivity analysis and obtained similar results (Supplementary Table S3).

During the 25 years covered by the survey, household income typically grew rapidly, and there is more intra-household variation in income over time than inter-household variation. We therefore use a wave-specific measure of household income, by dividing respondents into tertiles according to the positions of their households in the income distribution in each wave of the survey.

Control variables: Control variables include being an ethnic minority, being married, whether the respondent was working and whether they were residing in rural areas, and dummy variables representing the provinces in which the respondents resided.

### 2.2. Data Analysis

We apply grow-curve models, which have been commonly used in past studies to discern cohort variations and age trajectories [24,25]. First, we use linear growth-curve models with robust standard errors to model the number of cigarettes smoked per day, which take the following form:

(1) $$y_{ti}=B_{0}+SES_{ti}+Cohort_{i}\times SES_{ti}+Cohort_{i}^{2}\times SES_{ti}+Age_{ti}\times SES_{ti}+Age_{ti}\times Cohort_{i}\times SES_{ti}+Age_{ti}+\;Age_{ti}^{2}+Cohort_{i}+Cohort_{i}^{2}+Cohort_{i}^{3}+Cohort_{i}^{4}+Age_{ti}\times Cohort_{i}+Age_{ti}\times Cohort_{i}^{2}+Z_{lti}+u_{i}+\;v_{i}\times Age_{ti}+w_{ti}$$

$y_{ti}$ is the number of cigarettes smoked per day for individual *i* at time *t*. $SES_{ti}$ represents the set of indicators of SES status for individual *i*. A series of interaction terms examine how socioeconomic gaps in smoking change over time: $Cohort_{i}\times SES_{it}$ and $Cohort_{i}^{2}\times SES_{ti}$ capture how socioeconomic gaps in smoking change over cohorts; $Age_{ti}\times SES_{ti}$ assess how socioeconomic gaps in smoking change as individuals age within cohorts. Age, cohort, and their interaction term account for changes in smoking behavior over the life course and across cohorts. Higher-order polynomials of age and cohort are included to adequately model the relationship between age and cohort and the outcome variables For the sake of parsimony, higher-order polynomials are incrementally tested and kept in the models only if they are statistically significant. $Z_{lti}$ are a set of control variables. Random intercept and coefficient, $u_{i}$ and $v_{i}\times Age$, account for the clustering of repeated observations within individuals [29].

To model the other measure of smoking behavior, current smoking, we use logistic growth-curve models, which includes the same independent variables as in Equation (1).

## 3. Results

### 3.1. Descriptive Statistics of Dependent and Independent Variables

Table 1A displays the time-constant characteristics of the respondents. For covariates that changed with time, Table 1B displays both the time-constant and time-varying components. The overall rate of smoking was high: 68% of men reported being a smoker for at least one wave of the survey; the average male smoking rate for the overall sample is 58%, and on average, men smoked 9.5 cigarettes per day. In terms of socioeconomic characteristics, the percentages of men in the categories of high, medium and low levels of education are 33%, 43% and 24%. In any given wave of the survey, about one-third of men fell into each tertile of the income distribution.

### 3.2. Cohort Differences in Smoking among Men

**Figure 1:**
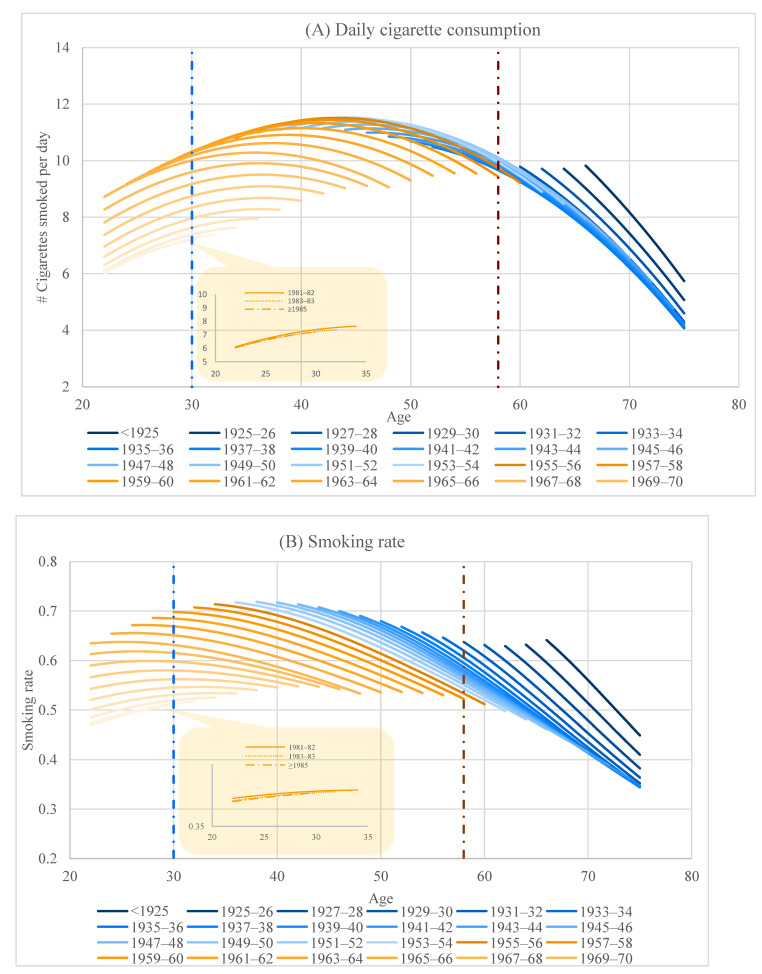
Life-course trajectories of smoking behavior by cohorts. Note 1: The predication is based on Models 1 and 4 in Table 2. Note 2: Vertical lines are placed at two age points, 30 and 58, to facilitate discerning changes in smoking across cohorts. The age points were chosen to maximize the cohorts they intersect with.

We first assess the overall changes in men’s smoking behavior by graphing the age trajectories of smoking of different cohorts, regardless of SES. Panels A and B in Figure 1 graph the estimated daily cigarette consumption and rate of smoking over the age range covered by the survey for each of the 32 cohorts; the estimates are based on Models 1 and 4 in Table 2. Age trajectories of smoking behavior of the oldest to youngest cohorts are arrayed in the figures from right to left. Consistent with previous studies [26], smoking among Chinese men increased with age when individuals were in their 20s and 30s, peaked in their 40s, and declined afterwards as they age.

As smoking behavior changes over age, when we examine how smoking varies across cohorts, we need to compare different cohorts at fixed age point(s). We use two age points, 30 and 58, and place a vertical line at each point to facilitate discerning changes in smoking across cohorts. The age points were chosen to maximize the cohorts they intersect with. The vertical blue line, placed at age 30, intersects with the cohorts born after 1960; the age trajectories of both cigarette consumption and probability of smoking of the cohorts born between 1960 and 1980 moved down steadily, indicating that smoking declined consistently across these cohorts. However, the decline stopped among the cohorts born after 1980; we amplified the graph for the three post-1980 cohorts to show that the age trajectories of these cohorts largely overlapped. The vertical brown line, placed at age 58, intersects with the cohorts born between 1930 and 1960. The age trajectories of daily cigarette consumption of these cohorts largely overlapped and those of smoking rate moved down slowly, indicating that smoking behavior did not change much for these cohorts. We do not focus on the cohorts born before 1930, because these cohorts existed the survey before 2005 and did not contribute to the changes in smoking behavior after 2005, when the decline of smoking started to stall. To sum, smoking behavior started to decline steadily in cohorts born between 1960 and 1980 but the decline stalled among the most recent cohorts, i.e., those born after 1980.

### 3.3. Changes in Socioeconomic Gaps in Smoking across Cohorts

We then examine the overall socioeconomic gaps in smoking (Models 1 and 4 in Table 2). High-education men smoked less than medium-education men, who in turn smoked less than low-education men. Compared with education, the relationship between household income and smoking is much weaker; only highest-income men were significantly less likely to be active smokers than lowest-income men (Model 4).

Next, we assess how socioeconomic gaps in smoking changed across cohorts, by adding interaction terms between cohort and SES indicators (Models 2 and 5 in Table 2); then interaction terms between cohort squared and SES indicators are added to capture the nonlinear changes (Models 3 and 6 in Table 2). Model 2 shows that educational gaps in cigarette consumption consistently expanded from early to recent cohorts, as indicated by the negative and significant interaction terms between cohort and the education dummies. The gap in cigarette consumption between high- and low-education men grew larger at a faster rate than that between medium-education and low-education men, as the interaction term between cohort and high education is significantly larger in magnitude than that between cohort and medium education (*p* = 0.007). In Model 3, the interaction terms between cohort squared and the education dummies are not significant, implying that educational gaps in cigarette consumption expanded largely in a linear fashion from early to recent cohorts. In terms of current smoking, Model 6 shows that educational gaps in the rate of smoking first narrowed in early cohorts and then grew bigger at an accelerated rate among recent cohorts, as the interaction terms between cohort and education dummies are positive whereas those between cohort squared and education dummies are negative and significant. Taken together, these findings show that educational gaps in smoking widened from older to younger cohorts.

**Figure 2:**
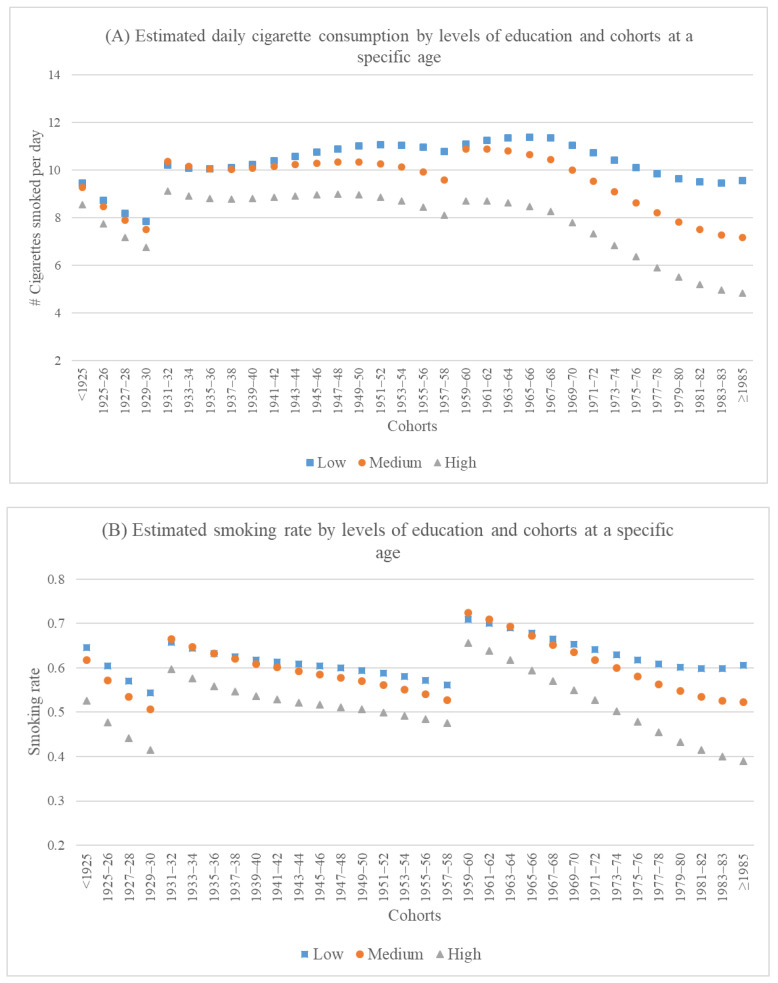
Cohort differences in educational gaps in smoking behavior. Note 1: The prediction is based on Models 3 and 6 in Table 2. Note 2: We display estimated smoking behavior at a specific age so as to present inter-cohort changes in educational gaps in smoking holding age constant. However, since the study period (1991–2015) covers different age ranges for different cohorts, we cannot find a single age that is covered by the study period for all cohorts. We therefore displayed estimated smoking behavior at 30 years for cohorts born on or after 1959, 58 years for those born between 1931 and 1958, and 68 years for those born on or before 1930. The age points are chosen to maximize the cohorts they intersect with.

Figure 2 visualizes the above findings regarding changing educational gaps across cohorts. It displays predicted daily cigarette consumption (Panels A) and rate of smoking (Panels B) at a specific age for men with different levels of education in all 32 cohorts. The prediction is based on Models 3 and 6 in Table 2. Both panels reveal that among those born after 1960, smoking behavior declined from older to younger cohorts, but the decline was steeper in men with higher levels of education than those with low education, leading to widening educational gaps. Most importantly, among the post-1980 cohorts, the declining trend stalled and possibly reversed for low-education men, but continued for those with medium or high education. As a result, the divergence in smoking behavior among men with different education levels became the starkest among these cohorts. This study is the first to identify widening inter-cohort educational gaps in smoking, which became especially amplified among the post-1980 cohorts.

In contrast to educational gaps in smoking, income gaps, to the extent that they exist, did not vary by cohort (Models 2, 3, 5 and 6 in Table 2).

### 3.4. Changes in Socioeconomic Gaps in Smoking over Age

We further assess whether socioeconomic differentials in smoking behavior changed over age for men. Results show that the gaps in smoking behavior, measured by both smoking rate and daily cigarette consumption, between medium- and low-education men expanded with age, as indicated by the negative and significant interaction terms between medium education and age in Models 3 and 6 in Table 2. When we compare high- and low-education men, we find the gap in one indicator of smoking, i.e., smoking rate, widened significantly over age, as indicated by the negative and significant interaction term between high education and age in Model 6; the gap in the other indicator, cigarette consumption, did not change significantly over age (Model 3). Taken together, the findings suggest that in general, educational gaps in smoking widened as men aged, implying that over the life course, more highly educated men tended to reduce smoking behavior at a faster rate than low-education men.

To visualize the changes of educational gaps over age within cohorts discussed above, in Supplementary Figure S1, we graph the educational gaps in daily cigarette consumption (Panels A and B) and rate of smoking (Panels C and D) over age for eight select cohorts. The graphs show that educational gaps in smoking increased with age, especially when medium-education men are contrasted with low-education men (Panels B and D). Moreover, in all graphs, the lines representing the youngest cohorts were higher than those of older cohorts, indicating expanding educational gaps across cohorts.

Our analyses show that income gaps also do not vary with age (Models 3 and 6 in Table 2).

### 3.5. Sensitivity Analyses

To ensure the robustness of the findings, we use dichotomized variables indicating whether men consumed 10/20 or more cigarettes per day as alternative measurements of the dependent variable. We also test an alternative measurement of education. We use a time-constant indicators of education (primary, secondary and tertiary in all cohorts), instead of the cohort-specific indicator of education in the main analyses. The results are consistent with those from the main analysis and are presented in Supplementary Tables S2 and S3.

## 4. Discussion

This paper extends previous research on social disparities in smoking in China, where high rates of male smoking have been a serious public health problem and smoking has important social and cultural meanings. We incorporate the life course approach and analyze longitudinal data to assess how socioeconomic gaps in smoking unfolded across age and cohorts.

We first confirmed that smoking participation declined from older to younger cohorts for those born after 1960 but the decline stalled in those born after 1980 [5]. We further showed that this stagnation was not uniformly distributed among groups with different SES. Among the post-1980 cohorts, for low-education men, the decline in smoking participation stopped and possibly reversed, whereas for men with higher levels of education, smoking participation continued to decline. The diverging trends led to the drastic increase in educational differential in smoking behavior among the most recent cohorts and to the stagnation in the overall trend of decline in smoking. Our study is the first to show the alarming trend that smoking behavior for low-education men stopped declining among the most recent cohorts. Future research needs to continue to monitor the smoking behavior of the youngest cohorts, paying special attention to those with low levels of education.

We also observed that the widening gaps in smoking between men of different levels of education among the post-1980 cohorts are a continuation and amplification of a long-term trend of expanding educational differentials that emerged among the cohorts born after 1960. In addition, our findings showed that within cohorts, educational gaps in smoking widened as men aged, especially when we contrasted medium-education men with low-education men. These findings on expanding inter- and intra-cohort educational gaps in smoking are consistent with the prediction of the theory of the fundamental causes of health. In China, although smoking serves important social functions and has significant cultural meanings among all social strata, individuals with more education were still able to avoid harmful health behavior and adopt healthful lifestyle at a faster pace than their more disadvantaged counterparts. These patterns are similar to those observed in many western social contexts, but with one crucial difference. That is, whereas in the west, smoking participation typically stabilized at a low level after a long decline [7], in China, the decline seemed to have stalled when smoking participation was still very widespread, driven by the stagnation and possible reversal that took place mainly among low-education men. More research is urgently needed to ascertain the causes of the stagnation. In particular, previous studies have shown that risk behaviors, educational attainment and labor market outcomes interact with one another during early adulthood, and family background and social context shaped these outcomes and the complex interaction among them [30,31,32,33]. It is possible that as China experienced industrialization and marketization, education became more important on the Chinese labor market [34] and low-education men became increasingly more disadvantaged, which contributed to their higher likelihood of smoking participation. Further studies will need to assess how early-life circumstances affect risk behavior and educational and labor-market outcomes, as well as how the patterns of influence evolved over cohorts.

We assessed two indicators of socioeconomic status and only found significant educational differentials in smoking, whereas household income was not an important predictor. This finding is consistent with a number of previous studies, which found education to be a more significant predictor of health behavior than income [35]. It is possible that cognitive abilities and knowledge are more influential to health behavior than access to material resources.

## 5. Conclusions

We identified a long-term widening in educational gaps in smoking in China. Our findings have important implications for social disparities of health in China. Given the harmful effects of smoking on a range of health outcomes [36,37], the widening educational gaps in smoking behavior across and within cohorts imply that social disparities in men’s health outcomes in later life may also expand. This study identified social groups that are vulnerable to smoking. It is essential for both scholars and policymakers to understand the pathways linking education to smoking initiation and cessation in contemporary China and develop interventions that target vulnerable groups.

## Figures and Tables

**Table 1:** (**A**) Time-invariant variables (N = 11,810); (**B**) Time-varying variables (N = 38,072 person-year observations).

(A)	
	%
Birth years ^1^	
<1935	8
1935–44	11
1945–54	19
1955–64	21
1965–74	23
1975–84	12
≥1985	5
Education	
Low	24
Medium	42
High	33
Rural	59
Ethnic minority	10
Province of residence	
Beijing	8
Liaoning	5
Helongjiang	6
Shanghai	8
Jiangsu	11
Shandong	11
Henan	11
Hubei	12
Hunan	11
Guangxi	7
Guizhou	5
Chongqing	5
(**B**)	
	% or Mean (SD)
Smoking	
Smoked at any given wave	58
# Cigarettes at any given wave (mean (SD))	9.5 (11)
Household income	
Bottom tertile at any given wave	32
Middle tertile at any given wave	34
Top tertile at any given wave	34
Married at any given wave	87
Working at any given wave	74

^1^. In the analysis, the sample is divided into 32 birth cohorts. In order to make the table readable, we combine the birth cohorts into 10-year intervals.

**Table 2:** Inter- and intra-cohort changes of socioeconomic gaps in smoking behavior.

	Daily Cigarette Consumption			Current Smoking		
	Model 1	Model 2	Model 3	Model 4	Model 5	Model 6
Education (reference: low)						
Medium	−0.875 ***	0.793	−0.291	−0.219 **	0.232	−0.397
High	−2.676 ***	−0.143	−0.746	−0.922 ***	−0.318	−1.353 ***
Household income (reference: lowest tertile)						
Middle tertile	0.095	−0.232	−0.271	−0.046	−0.267	−0.420
Top tertile	−0.159	0.132	−0.129	−0.183 ***	−0.247	−0.387
Cohort × SES						
Cohort × medium education		−0.070 **	0.095		−0.015	0.083
Cohort × high education		−0.129 ***	−0.037		−0.027 **	0.134 **
Cohort^2^ × medium education			−0.005			−0.003 **
Cohort^2^ × high education			−0.003			−0.005 ***
Cohort × middle terile income		0.003	0.009		0.009	0.033
Cohort × top tertile income		−0.025	0.014		0.003	0.024
Cohort^2^ × middle terile income			−0.0002			−0.001
Cohort^2^ × high tertile income			−0.001			−0.001
SES × age						
Age × medium education		−0.036 *	−0.042 *		−0.018 **	−0.022 **
Age × high education		−0.013	−0.017		−0.012	−0.019 *
Age × middle tertile income		0.021	0.021		0.005	0.004
Age × top tertile income		0.013	0.010		0.002	0.0000

*** *p* < 0.001; ** *p* < 0.01; * *p* < 0.05; two-tailed test. Note: Models 1–3 are based on linear random-coefficient models and Models 4–6 are based on logistic random-coefficient models. All models control for age, cohort, higher-order polynomials of age and cohort, interaction terms between age and cohort, marital status, ethnic minority, currently work, rural residence, and province of residence. The results for the control variables are shown in Supplementary Table S1.

## Data Availability

The research is based on the data from the Chinese Health and Nutrition Survey, which are publicly available at https://www.cpc.unc.edu/projects/china (accessed on 5 April 2022).

## Supplementary Materials

The following supporting information can be downloaded at: https://www.mdpi.com/article/10.3390/ijerph19084917/s1, Text S1. Cohort-constant and cohort-specific measures of low, medium and high levels of education; Table S1. Results for the control variables in models presented in Table 2; Figure S1. Educational differences in smoking behavior over age in select cohorts; Table S2. Sensitivity analysis: being a heavy smoker as the dependent variable; Table S3. sensitivity analysis: using cohort-constant measure of levels of education.
