# Supplementary material for: Diverging Trends and Expanding Educational Gaps in Smoking in China
Source: Int J Environ Res Public Health. 2022 Apr 18;19(8):4917. doi: 10.3390/ijerph19084917 (PMC9033051; doi:10.3390/ijerph19084917)
Supplement: Supplementary file 1 [file ijerph-19-04917-s001.zip › ijerph-1529094-supplementary.pdf]

Supplementary Materials

Text S1. Cohort-constant and cohort-specific measures of low, medium and high levels of education.

Educational opportunities expanded tremendously across the cohorts in this study. If we apply the same definition of low, medium and high levels of education to all cohorts (i.e. defining primary education or less (0-6 years) as low, some junior high and high school education (7-11 years) as medium, and high school or more (≥12 years) as high), the distribution of levels of education is highly unbalanced across cohorts. The following table displays the percentages in each level of education in the overall sample and 8 select cohorts among men. It shows the highly unbalanced education levels across cohorts; for example, the percent of men having low level of education (0-6 years) is 73% in the 1929-30 cohort and 3.6% in the post-1985 cohort.

Levels of education	Years of education	Whole sample %	Birth years 1929-30 %	1937-38 %	1945-46 %	1953-54 %	1961-62 %	1969-70 %	1977-78 %	≥1985 %
Low	0-6	31	73	60	52	36	10	18	8.8	3.6
Medium	7-11	40	14	19	29	43	48	47	50	39
High	≥12	28	13	20	19	21	42	35	42	57

In the main analysis, we therefore used a cohort-specific measure of low, medium and high levels of education so that the distribution of levels of education is roughly balanced across cohorts. We also selected the cutoff points such that they correspond to meaningful education levels (e.g. primary school and high school). The following table shows the percentages in each level of education in the overall sample, the cohort-specific definition of education levels, and distribution across cohorts:

Whole sample		Birth years <1935		Birth years 1935-38		Birth years 1938-54		Birth years 1955-78		Birth years >1978	
Levels of education	%	Years of education	%	Years of education	%	Years of education	%	Years of education	%	Years of education	%
Low	27	0	28	0-3	33	0-5	30	0-8	23	0-9	43
Medium	44	1-6	44	4-8	33	6-9	41	9-11	44	10-12	31
High	29	> 6	28	>8	35	>9	29	>11	33	>12	29

The distributions of cohort-constant and cohort-specific measures of low, medium and high levels of education in the overall sample are quite similar, but the distribution of cohort-specific measure across cohorts is much more balanced than that of the cohort-constant measure. We also performed analysis with the cohort-constant measure in sensitivity analyses.

Table S1. Results for the control variables in models presented in Table 2.

	Daily cigarette consumption			Current smoking		
	Model 1	Model 2	Model 3	Model 4	Model 5	Model 6
Control variables						
Age	-0.429***	-0.416***	-0.414***	-0.169***	-0.163***	-0.158***
	(0.049)	(0.051)	(0.051)	(0.021)	(0.022)	(0.022)
Age^2	-0.008***	-0.008***	-0.008***	-0.002***	-0.002***	-0.002***
	(0.001)	(0.001)	(0.001)	(0.0004)	(0.0004)	(0.0004)
Cohort	-0.304	-0.271	-0.356	-0.180	-0.173	-0.241*
	(0.227)	(0.228)	(0.235)	(0.102)	(0.102)	(0.105)
$Cohort^2$	0.076**	0.080**	0.080**	0.038***	0.039***	0.038***
	(0.027)	(0.027)	(0.027)	(0.012)	(0.012)	(0.012)
$Cohort^3$	-0.004***	-0.005***	-0.004***	-0.002*	-0.002*	-0.002*
	(0.001)	(0.001)	(0.001)	(0.001)	(0.001)	(0.001)
$Cohort^4$	0.0001***	0.0001***	0.0001***	0.00003***	0.00003***	0.00003***
	(0.00002)	(0.00002)	(0.00002)	(0.00001)	(0.00001)	(0.00001)
Age × cohort	0.050***	0.050***	0.050***	0.011***	0.011***	0.011***
	(0.006)	(0.006)	(0.006)	(0.003)	(0.003)	(0.003)
Age × $cohort^2$	-0.001***	-0.001***	-0.001***	-0.0001	-0.0001	-0.0001
	(0.0002)	(0.0002)	(0.0002)	(0.0001)	(0.0001)	(0.0001)
Married	0.793***	0.727***	0.723***	0.327***	0.318***	0.311***
	(0.177)	(0.178)	(0.178)	(0.075)	(0.076)	(0.076)
Ethnic minority	-0.395	-0.388	-0.383	-0.137	-0.136	-0.134
	(0.293)	(0.292)	(0.292)	(0.126)	(0.127)	(0.127)
Currently working	1.207***	1.249***	1.248***	0.396***	0.399***	0.397***
	(0.137)	(0.138)	(0.138)	(0.055)	(0.055)	(0.055)
Living in rural areas	0.704***	0.686***	0.683***	0.100	0.095	0.092
	(0.169)	(0.168)	(0.168)	(0.073)	(0.073)	(0.073)

Table S1. Results for the control variables in models presented in Table 2, continued.

	Daily cigarette consumption			Current smoking		
	Model 1	Model 2	Model 3	Model 4	Model 5	Model 6
Province						
Liaoning	1.503***	1.279***	1.272***	0.453*	0.396	0.367
	(0.438)	(0.442)	(0.442)	(0.219)	(0.222)	(0.222)
Helongjiang	-0.337	-0.523	-0.535	-0.176	-0.226	-0.265
	(0.431)	(0.435)	(0.435)	(0.222)	(0.224)	(0.224)
Shanghai	0.492	0.460	0.467	0.255	0.247	0.243
	(0.447)	(0.446)	(0.446)	(0.249)	(0.249)	(0.249)
Jiangsu	0.248	0.139	0.137	0.120	0.083	0.060
	(0.423)	(0.427)	(0.427)	(0.215)	(0.217)	(0.217)
Shangdong	0.178	0.0938	0.0805	-0.095	-0.127	-0.164
	(0.418)	(0.421)	(0.421)	(0.216)	(0.218)	(0.218)
Henan	1.303***	1.170***	1.161***	0.475*	0.438*	0.397
	(0.428)	(0.432)	(0.432)	(0.215)	(0.217)	(0.217)
Hubei	1.769***	1.651***	1.644***	0.127	0.088	0.052
	(0.450)	(0.452)	(0.452)	(0.218)	(0.219)	(0.219)
Hunan	4.281***	4.149***	4.155***	0.657***	0.616**	0.592**
	(0.452)	(0.454)	(0.454)	(0.216)	(0.217)	(0.218)
Guangxi	1.051**	0.844*	0.845*	0.070	0.015	-0.010
	(0.430)	(0.433)	(0.434)	(0.212)	(0.215)	(0.215)
Guizhou	3.163***	2.999***	2.987***	1.510***	1.466***	1.433***
	(0.437)	(0.439)	(0.439)	(0.224)	(0.225)	(0.226)
Chongqing	2.064***	1.921***	1.919***	1.012***	0.965***	0.943***
	(0.523)	(0.526)	(0.526)	(0.269)	(0.271)	(0.271)
Constant	7.805***	6.679***	7.364***	0.741*	0.540	1.153**
	(0.767)	(0.834)	(0.927)	(0.361)	(0.384)	(0.427)
Observations	37,870	37,870	37,870	38,223	38,223	38,223
Number of groups	11,797	11,797	11,797	11,818	11,818	11,818

Standard errors in parentheses

*** *p<0.001*, ** *p<0.01*, * *p<0.05*

Note: Models 1-4 are based on linear random-coefficient models and Models 5-8 are based on logistic random-coefficient models.

Figure S1. Educational differences in smoking behavior over age in select cohorts.

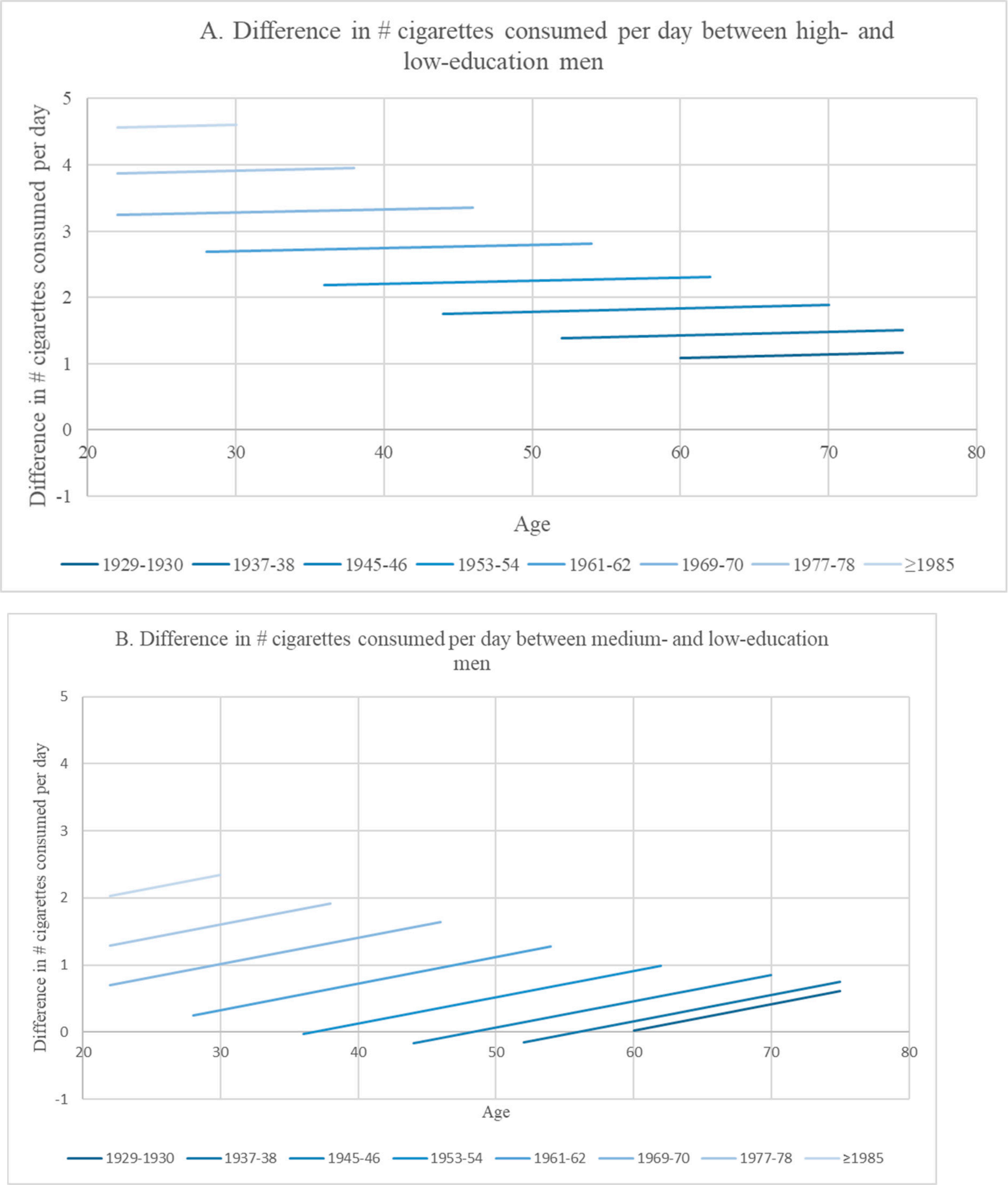

Figure S1. Educational differences in smoking behavior over age in select cohorts, continued.

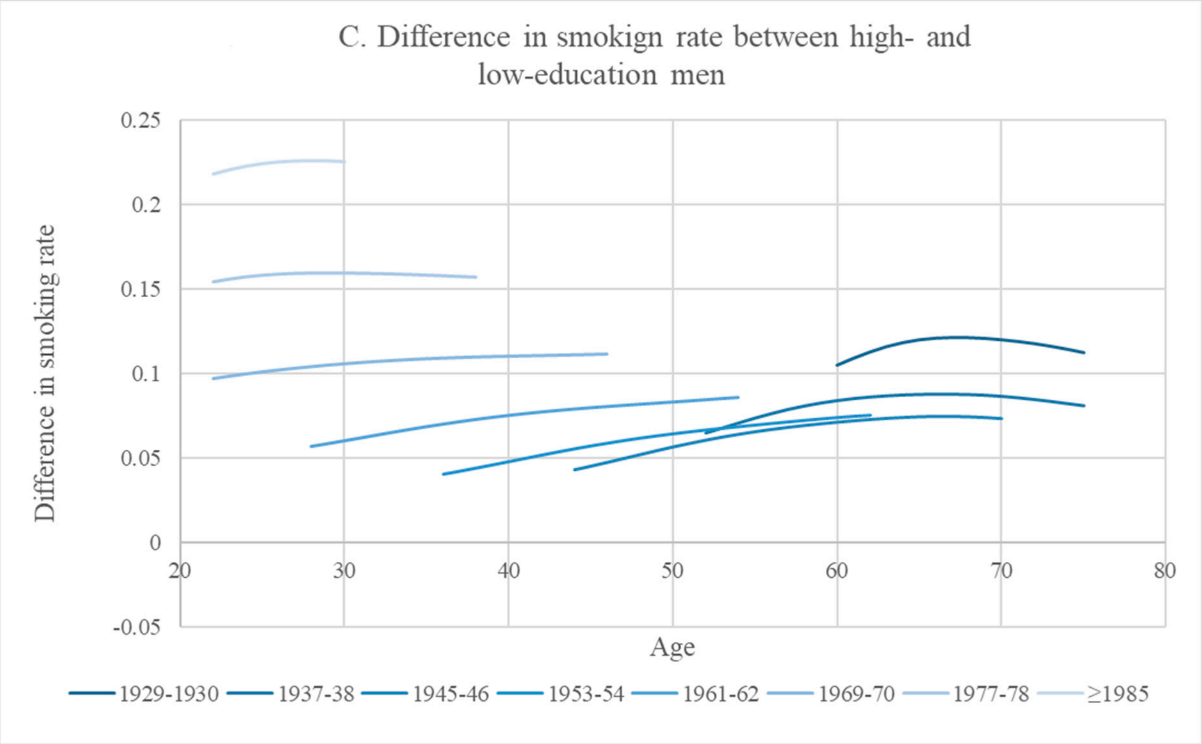

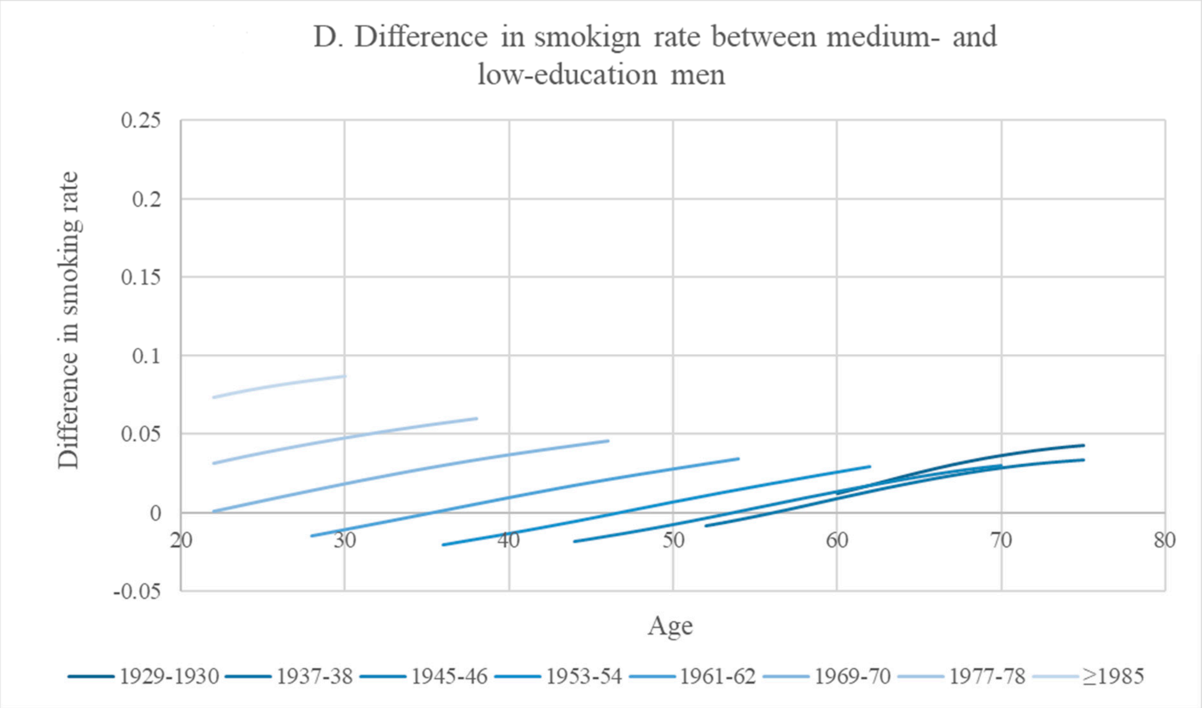

Note 1: The predication is based on Models 3 and 6 in Table 2.

Table S2. Sensitivity analysis: being a heavy smoker as the dependent variable.

	Being a heavy smoker		
	Model 1	Model 2	Model 3
Education (reference: low)			
Medium	-0.281***<br>(0.070)	0.329<br>(0.187)	0.096<br>(0.302)
High	-0.946***<br>(0.082)	0.253<br>(0.215)	-0.289<br>(0.345)
Household income (reference: lowest tertile)			
Middle tertile	0.002<br>(0.0435)	-0.049<br>(0.141)	0.128<br>(0.230)
Top tertile	-0.067<br>(0.0482)	0.130<br>(0.150)	0.119<br>(0.245)
Cohort × SES			
Cohort × medium education		-0.028***<br>(0.009)	0.007<br>(0.037)
Cohort × high education		-0.059***<br>(0.010)	0.024<br>(0.043)
$Cohort^2$ × medium education			-0.001<br>(0.001)
$Cohort^2$ × high education			-<br>0.002**<br>(0.001)
Cohort × middle terile income		-0.002<br>(0.007)	-0.029<br>(0.029)
Cohort × top tertile income		-0.015*<br>(0.007)	-0.015<br>(0.031)

	Being a heavy smoker		
	Model 1	Model 2	Model 3
SES × age			
Age × medium education		-0.010<br>(0.006)	-0.011<br>(0.007)
Age × high education		-0.012<br>(0.008)	-0.015*<br>(0.008)
Age × medium education × cohort			
Age × high education × cohort			
Age × middle tertile income		0.007<br>(0.006)	0.008<br>(0.006)
Age × top tertile income		0.006<br>(0.006)	0.006<br>(0.006)

Cohort^2 × middle terile income	0.001<br>(0.001)	Observations	37,870	37,870	37,870
Cohort^2 × high tertile income	0.00002<br>(0.001)	Number of groups	11,797	11,797	11,797

Standard errors in parentheses

**** p<0.001, ** p<0.01, * p<0.05*

Note: Based on logistic random-coefficient models. All models control for age, cohort, higher-order polynomials of age and cohort, interaction terms between age and cohort, marital status, ethnic minority, currently work, rural residence, and province of residence.

Table S3. sensitivity analysis: using cohort-constant measure of levels of education.

	Daily cigarette consumption			Current smoking		
	Model 1	Model 2	Model 3	Model 4	Model 5	Model 6
Education (reference: low)						
Medium	-.548*	-0.079	-0.096	-0.157	-0.4	-0.398
	(.22)	(.58)	(.906)	(.091)	(.24)	(.394)
High	-2.588***	-0.994	-1.663	-.865***	-.965***	-1.945***
	(.242)	(.654)	(.986)	(.103)	(.28)	(.475)
Household income (reference: lowest tertile)						
Middle tertile	0.087	-0.214	-0.27	-0.05	-0.251	-0.423
	(.113)	(.363)	(.522)	(.048)	(.153)	(.234)
Top tertile	-0.165	0.185	-0.099	-.188***	-0.21	-0.362
	(.127)	(.394)	(.589)	(.053)	(.165)	(.256)
Cohort × SES						
Cohort × medium education		-0.022	-0.008		0.023	0.037
		(.029)	(.119)		(.013)	(.051)
Cohort × high education		-.085**	0.01		0.015	.152**
		(.031)	(.125)		(.014)	(.058)
Cohort^2 × medium education			-0.001			-0.001
			(.004)			(.002)
Cohort^2 × high education			-0.003			-.004*
			(.004)			(.002)
Cohort × middle terile income		0.001	0.011		0.007	0.035
		(.016)	(.066)		(.007)	(.03)
Cohort × top tertile income		-0.029	0.016		0.00002	0.025
		(.017)	(.074)		(.007)	(.032)
Cohort^2 × middle terile income			-0.0003			-0.001
			(.002)			(.001)
Cohort^2 × high tertile income			-0.001			-0.001
			(.002)			(.001)

Table S3. sensitivity analysis: using cohort-constant measure of levels of education, continued.

	Daily cigarette consumption			Current smoking		
	Model 1	Model 2	Model 3	Model 4	Model 5	Model 6
SES × age						
Age × medium education		-0.024	-0.024		-0.013	-0.013
		(.018)	(.019)		(.008)	(.008)
Age × high education		-0.006	-0.009		-0.011	-0.015
		(.02)	(.021)		(.009)	(.009)
Age × medium education × cohort						
Age × high education × cohort						
Age × middle tertile income		0.022	0.021		0.006	0.004
		(.014)	(.015)		(.006)	(.007)
Age × top tertile income		0.014	0.01		0.002	0.0002
		(.015)	(.016)		(.007)	(.007)
Observations	38,223	38,223	38,223	37,870	37,870	37,870
Number of groups	11,818	11,818	11,818	11,797	11,797	11,797

Standard errors in parentheses

*** $p<0.001$, ** $p<0.01$, * $p<0.05$

Note: Models 1-3 are based on linear random-coefficient models and Models 4-6 are based on logistic random-coefficient models. All models control for age, cohort, higher-order polynomials of age and cohort, interaction terms between age and cohort, marital status, ethnic minority, currently work, rural residence, and province of residence.
